# Estimating the magnitude and sensitivity of energy fluxes for stickleback hosts and *Schistocephalus solidus* parasites using the metabolic theory of ecology

**DOI:** 10.1002/ece3.10755

**Published:** 2023-12-03

**Authors:** Danielle C. Claar, Sara M. Faiad, Natalie C. Mastick, Rachel L. Welicky, Maureen A. Williams, Kristofer T. Sasser, Jesse N. Weber, Chelsea L. Wood

**Affiliations:** ^1^ Washington State Department of Natural Resources Olympia Washington USA; ^2^ School of Aquatic and Fishery Sciences University of Washington Seattle Washington USA; ^3^ Unit for Environmental Sciences and Management North‐West University Potchefstroom South Africa; ^4^ College of Arts and Sciences Neumann University Aston Pennsylvania USA; ^5^ Biology Department McDaniel College Westminster Maryland USA; ^6^ Department of Biological Sciences University of Alaska Anchorage Anchorage Alaska USA; ^7^ Department of Integrative Biology University of Wisconsin Madison Madison Wisconsin USA

**Keywords:** energy flux, *Gasterosteus aculeatus*, MTE, parasite, respirometry

## Abstract

Parasites are ubiquitous, yet their effects on hosts are difficult to quantify and generalize across ecosystems. One promising metric of parasitic impact uses the metabolic theory of ecology (MTE) to calculate energy flux, an estimate of energy lost to parasites. We investigated the feasibility of using metabolic scaling rules to compare the energetic burden of parasitism among individuals. Specifically, we found substantial sensitivity of energy flux estimates to input parameters used in the MTE equation when using available data from a model host–parasite system (*Gasterosteus aculeatus* and *Schistocephalus solidus*). Using literature values, size data from parasitized wild fish, and a respirometry experiment, we estimate that a single *S. solidus* tapeworm may extract up to 32% of its stickleback host's baseline metabolic energy requirement, and that parasites in multiple infections may collectively extract up to 46%. The amount of energy siphoned from stickleback to tapeworms is large but did not instigate an increase in respiration rate in the current study. This emphasizes the importance of future work focusing on how parasites influence ecosystem energetics. The approach of using the MTE to calculate energy flux provides great promise as a quantitative foundation for such estimates and provides a more concrete metric of parasite impact on hosts than parasite abundance alone.

## Graphical abstract

While parasites are ubiquitous across ecosystems, their effects on host energy flow (and thus broader ecological implications) are difficult to quantify and generalize across species. In this study, we estimated host–parasite energy flux to build upon fundamental ecological theory and develop capacity for utilizing this common‐currency approach across ecosystems.

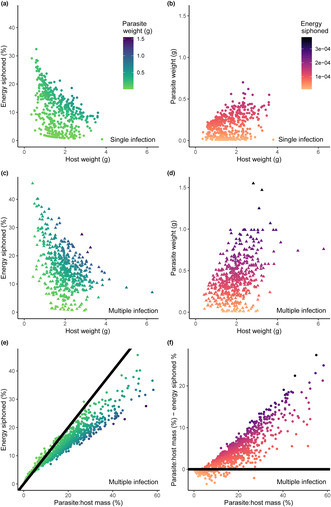

## 1 INTRODUCTION

Ecologists estimate energy fluxes through ecosystems to quantify and predict everything from food web structure to population dynamics. But most models of energy flux exclude an important energy pool: parasites. Parasitism is ubiquitous across taxa, ecosystems, and biomes; it has been estimated that 40% of all species are parasites (Dobson et al., 2008). Parasite energy use is an often‐unseen drain on host energy because of their generally small individual body size. However, at the ecosystem level, parasite standing‐stock biomass can be substantial, matching or exceeding the biomass of important groups of free‐living organisms, and therefore driving population‐ and ecosystem‐level dynamics (Kuris et al., 2008). In addition to directly taking energy from host production (i.e., energy siphoning, defined as energy flux from host to parasite), parasites mediate energy fluxes by modifying host physiology and behavior (Barber et al., 2000, 2008) and by serving as prey themselves (Lafferty et al., 2008). Although the effects of parasitism on a system can vary by host and parasite lifestyle and life stage (Altman et al., 2016; Thompson et al., 2005), and the overall impact of parasitism has been measured via respirometry of the host (e.g., Chodkowski & Bernot, 2017; Lettini & Sukhdeo, 2010; Nadler et al., 2021), the direct energetic fluxes of parasitism are rarely measured (e.g., Hechinger, 2013).

Three approaches are available for comparing energy consumed by parasitism across host species, parasite species, life stage, and habitat. Directly measuring energy flux is often difficult in host–parasite systems, as the specialized environments provided to parasites by hosts are often not reproducible in vitro. Alternatively, using energy flux proxies (e.g., biomass) provides a coarse but easily measured estimate of the impact of parasites on ecosystems and hosts (Kuris et al., 2008; Mitchell, 2003; Paseka, 2017). However, there may be mismatches between biomass estimates and actual energy flux (Grunberg & Anderson, 2022). A third approach, which promises improved estimates, is to derive parasite energy flux from models of host and parasite energetic outcomes predicted by the metabolic theory of ecology (MTE; Table 1; e.g., Brown et al., 2004; Gillooly et al., 2001; Price et al., 2012). MTE calculations (and related scaling laws) have been used to estimate energy use and flux in free‐living systems (e.g., Marquet et al., 2004), and also to estimate the population dynamics of parasites alongside free‐living species in ecosystems (Cohen et al., 2016; Hechinger et al., 2011; Lagrue et al., 2014), as well as scaling relationships between host body mass and parasite density (Arneberg et al., 1998; George‐Nascimento et al., 2004) and parasite biomass (Poulin & George‐Nascimento, 2007). However, these calculations also hold potential for estimating the energy a parasite takes from its host (i.e., energy flux; Hechinger et al., 2012, 2019; Hechinger, 2013). One can calculate energy flux with an MTE scaling equation that incorporates assumptions about organismal metabolism with temperature and mass data that are easy to source for many organisms (Equation 1; Table 1; e.g., Brown et al., 2007; Clarke & Johnston, 1999; Clarke, 2006). In the past decade, the metabolic theory of ecology has been applied to assess various aspects of host–parasite models, including the thermal dependencies of parasites (Molnár et al., 2013), host–parasite dynamics under broad thermal gradients (Kirk et al., 2018, 2019; Rohr et al., 2013), and identifying the “optimal” size of a parasite species for a host of a given body size (De Leo et al., 2016). Given that this equation exists and is relatively simple to calculate, why has not it been widely adopted for studying parasitism?

**TABLE 1:** Explanation of energy flux equation and related variables (Hechinger, 2013).

Equation 1. $F_{x}=\left(iM_{x}^{\alpha_{x}}e^{-E/\mathrm{kT}}\right)N_{x}$	
*F*_*x*_	*Energy flux*. *F*_p_ represents energy flux of the parasite infrapopulation or infracommunity. *F*_p_ = *I*_p_ in single infections. *F*_h_ represents the energy flux of the host.
*I*_*x*_	*Individual metabolic rate*. The assimilation, synthesis, and use of energy by a single organism. The individual metabolic rate, *I*, is derived from $I_{x}=\left(iM_{x}^{\alpha_{x}}e^{-E/\mathrm{kT}}\right)$, where metabolic rate scales with body mass, *M*, and varies with temperature, *T*. The normalization constant is *i*, and $f\left(T\right)=e^{-E/\mathrm{kT}}$ is a function that represents how temperature influences metabolic rate.
*α*	*Scaling exponent*. The scaling exponent, *α*, is generally predicted to be about ¾ (Savage, 2004), and prior work quantifying parasite energy flux has utilized a scaling exponent of ¾ in energy‐limited relationships (Hechinger, 2013). However, the scaling exponent does vary across taxa; it may be closer to ⅔ for birds and mammals (White & Seymour, 2003) and >1 for unicellular organisms (De Long et al., 2010; Makarieva et al., 2008).
*E*	Activation energy of metabolism. This is the slope of the relationship $\ln\left(IM^{-3/4}\right)=-E\left(1/\mathrm{kT}\right)+\ln\left(i_{0}\right)$, which is the mass‐normalized metabolic rate as a function of 1/T (Brown et al., 2004; Gillooly et al., 2001). Most recent analyses have found this value to fall within 0.41–0.72 eV, with a mean of 0.63 eV for aerobic respiration (Gillooly et al., 2001). For previous studies involving parasites, 0.63 eV has been used for *E* (Brown et al., 2004; Hechinger, 2013), even though parasites are known to rely substantially on anaerobic metabolism (causing standard empirical scaling relationships to underestimate parasite energy flux; Hechinger et al., 2012).
*i*	Metabolic normalization constant. This is fit empirically, and varies for organisms of different physiological types (Brown et al., 2004; Clarke, 2006). It corrects for natural variability in metabolism (e.g., differing metabolic rates of ectotherms and endotherms). This variable is unitless.
*m*	*Mass*. The mass of the organism for which energy flux is calculated, typically measured in g or kg.
*k*	*Boltzmann constant*. A physical constant that relates the average kinetic energy of a particle with the temperature. *K* = 1.380649 × 10^−23^ J K^−1^ or 8.62 × 10^−5^ eV K^−1^.
*T*	*Temperature*. For ectotherms, this is the temperature of the surrounding environment; typically measured in K.
*N*	*Number of parasites*. The abundance or number of individual parasites residing within the host.

One reason is that little is known about which parameters (Equation 1; Table 1) are the most influential for host and parasite energy fluxes, leaving potential users in the dark about which parameters can reasonably be extracted from the literature (e.g., from similar taxa) and which need to be empirically determined. Although progress is being made in the development of this approach (e.g., Kirk et al., 2018, 2019; O'Connor & Bernhardt, 2018), there are additional considerations for using the MTE to calculate energy flux in host–parasite systems; specifically, which parameters can be assumed, and which parameters, if assumed incorrectly, would lead to the largest errors in estimates of energy flux? For example, normalization constants vary among organisms, and should be estimated for both the host and parasite to accurately calculate energy flux. Previous studies circumvented the uncertainty of normalization constants by using the MTE to calculate relative energy flux within a single species (i.e., assuming that the normalization constant remains the same) or by calculating relative energy flux for a wide range of species (i.e., assuming that variability cancels out on average) (Hechinger, 2013; Hechinger et al., 2019). This approach provides information about scaling relationships but not absolute energy fluxes. Consequently, relative energy flux measurements cannot be used to calculate the percentage (or absolute amount) of energy siphoned from the host without large amounts of uncertainty, and cannot be compared across host individuals, sites, or ecosystems. These potential pitfalls are not insurmountable but must be carefully considered if MTE is to be used broadly to calculate estimates of parasite energy flux from hosts.

Parasitologists and parasite ecologists generally quantify the number of individuals of a specific species of parasite within a host (i.e., abundance data; Poulin & Jorge, 2019). These data are not only widely available, but also can be leveraged to understand the ecological impacts of parasites in the context of MTE. For example, connecting abundance data with information on parasite and host traits could yield novel and impactful insights into the relative costs of parasitism (Hechinger, 2015; Hechinger et al., 2011). In this manuscript, we specifically focus on body mass scaling relationships and energy flux for individual species to estimate actual energy flux of parasites and their hosts. Because we recognize that individual species vary from the average MTE scaling and flux predictions (e.g., Brown et al., 2004; Gillooly et al., 2001), we use experiments to accurately estimate the empirical parameters of energy flux for taxa of interest. Moreover, we compare our empirical parameter estimates to those commonly used in MTE studies, particularly parameters that can be reasonably expected to differ among taxa, and test the extent to which inaccurate parameters may influence final estimates of energetic flux due to parasitism.

To explore the consequences of various analytical choices, we selected a fish model because, as ectotherms, their body temperature estimates can be obtained from measurements of water temperature. We further simplified the model by focusing on a parasite species that is easy to identify and measure at an individual scale. The three‐spined stickleback (*Gasterosteus aculeatus*) and its tapeworm parasite (*Schistocephalus solidus*; class Cestoda) are a well‐studied and widely used experimental system (Barber & Scharsack, 2010). The stickleback is the second intermediate host of the tapeworm (plerocercoid larval phase), a stepping stone in its complex life cycle between a cyclopoid copepod intermediate host and a piscivorous bird definitive host (Heins & Baker, 2008). *S. solidus* can grow to large sizes within their stickleback host: individual parasites reach up to 40% of host biomass (Hopkins & Smyth, 1951) and under multiple infections the total cestode mass can exceed that of the host fish (Barber, 2007). It is well documented that fish‐specific cestodes (Order Pseudophyllidea) such as *S. solidus* can impact maturation, swimming ability, reproduction, growth rate, and mortality of their hosts (Heins & Baker, 2008; Pascoe & Mattey, 1977; Pennycuick, 1971). The metabolic cost of *S. solidus* on threespine stickleback fish has been studied using various methods, including feeding and assimilation rates (Arnott et al., 2000; Barber, 2005; Wright et al., 2008) and respiration of migrating and non‐migrating individuals (Andrea et al., 2017; Lester, 1971; Meakins & Walkey, 1975). Overall, the stickleback–tapeworm system provides opportunities for MTE calculations of energy flux across many conditions.

Using both infection burden data (which incorporates both parasite abundance and mass) and respiration measurements from this host–parasite system, we: (1) ground‐truth theoretical estimates of energy flux for both infected and uninfected three‐spined stickleback with controlled lab‐based respiration measurements, (2) illustrate which MTE parameters have the strongest effect on flux estimates, and (3) demonstrate that a common method of simplifying MTE calculations (i.e., using mean parasite mass instead of individual worm measurements of mass) can substantially bias estimates of the energetic burden of infections involving multiple worm individuals when parasite body size is variable. These approaches allowed us to test the magnitude and sensitivity of energy flux estimates for stickleback hosts and tapeworm parasites, following Hechinger's (2013) approach to the metabolic theory of ecology.

## 2 METHODS

### 2.1 Sample information

To determine how much energy *S. solidus* is extracting from its host, *Gasterosteus aculeatus*, data were collected from three‐spine stickleback and *S. solidus* (Figure S1). Three datasets were synthesized: (1) *S. solidus*‐infected stickleback from Lake Iliamna, Alaska for addressing parasite size variability in infections involving multiple worm individuals (hereafter, “multiple infections”), (2) *S. solidus*‐infected stickleback from Lake Aleknagik, Alaska for realistic mass measurements of parasites and hosts in a natural population (Table S1), and (3) lab‐reared uninfected and *S. solidus*‐infected stickleback hybridized from two British Columbia locations (Gosling Lake and Sayward Estuary), for respirometry measurements to estimate MTE energy flux equation parameters *α* and *i*. The first two datasets were used together because the Lake Iliamna dataset did not represent a random sample of fish, and the Lake Aleknagik dataset did not include measurements of individual parasites, but did provide a large, random sample of *S. solidus* burden in the field. These datasets are described in Appendix S1.

### 2.2 Empirical determination of host energy flux using respirometry

The resting metabolic rates of individuals from a hybridized “population” of three‐spined stickleback composed of reciprocal crosses of two lab‐reared stickleback populations, from Gosling Lake (G) and Sayward (S) (British Columbia; 50.385, −125.951) *n* = 30 per crossed population were empirically estimated using intermittent‐flow respirometry and a static respirometer. Individuals were measured 42, 60, and 80 days after exposure to copepods infected with *S. solidus* (or sham exposed as a control) using randomized block scheduling. Given that the experimental setup consisted of 4 respirometry chambers and 20 fish per treatment, measurements were conducted over a 10‐day period using a randomized block design (Appendix S1). Standard operating procedures were followed for respiration measurements and for background, absolute, and mass specific metabolic rate calculations (Appendix S1; R package *FishResp*, Morozov et al., 2019).

First, differences in log‐transformed temperature‐corrected individual metabolic rates for infected and uninfected fish were tested separately using two linear models (R function *lm*), with a fixed effect of fish mass. MTE parameters were extracted, where the intercept of the fit represented the normalization constant (C = ln(*i*)), and the slope represented the scaling exponent (*α*). Second, a full linear model was constructed including fixed effects for fish mass, population, and infection status. To test whether the difference in sample size between infected (*n* = 12) and uninfected (*n* = 73) influenced the results, this model was also run using a balanced, randomized subset of the data, where *n* = 12 for both infected and uninfected fish.

### 2.3 Using MTE to estimate energy flux of *Schistocephalus solidus* in Alaskan sticklebacks

Energy flux was calculated using parameters from Table 2 for the Lake Aleknagik dataset. The energy flux of the stickleback host was calculated and compared to the energy flux of *S. solidus* to conservatively estimate the percent of energy extracted by parasites using

(2) $$\text{Minimum}\;\%\;\text{of host energy siphoned}\;\mathrm{by}\;\text{parasite}=\frac{\left(F_{p}\right)}{\left(F_{h}\right)}*100.$$

**TABLE 2:** Metabolic theory of ecology parameters used in this study, based on values from the literature, and values estimated in the current study.

Parameter		Stickleback	*Schistocephalus*
Normalization constant	C (*i*)	17.71 (49,130,963) (empirically estimated in this study)	17.17 (28,630,983) (Brown et al., 2004; invertebrates)
Scaling exponent	*α*	1.039 (empirically estimated in this study)	0.75 (Kleiber, 1932; theoretical value)
Activation energy for aerobic respiration	*E*	0.63 eV (Brown et al., 2004)	0.63 eV (Brown et al., 2004)
Temperature	*T*	15°C (288.15 K) (lake temperature chosen for this study)	15°C (288.15 K) (lake temperature chosen for this study)

Energy siphoning was plotted across host masses for single and multiple infections (Figure 4a–d). This calculation estimates the percentage of energy siphoning at the individual level. Mean energy flux of the population can then be estimated as the mean of individuals calculated from Equation (2). Alternatively, energy flux at the population level can be calculated as the slope of the linear model $F_{p}\sim \text{slope}*R_{h}$ (Grunberg & Anderson, 2022), where $F_{p}$ represents the energy flux of the parasite infrapopulation, and $\;R_{h}$ represents the whole host organism metabolic rate. We compare results from both the individual calculation and the population calculation.

The parasite‐to‐host mass ratio was calculated and compared to the proportional energy flux using

(3) $$\%\;\text{of host mass to parasite mass}=\frac{\left(M_{p}\right)}{\left(M_{h}\right)}*100.$$

Finally, the difference between parasite‐to‐host energy flux and mass was calculated by subtracting the result of Equation (3) from the result of Equation (2). The difference between mass ratios (Equation 3) and energy flux (Equation 2) ratios was calculated. A comparison of parasite‐to‐host mass and energy siphoning (i.e., energy flux) was also plotted for all hosts (Figure 4e) as well as the difference in mass and energy flux ratios across a range of mass ratios for all hosts (Figure 4f).

### 2.4 Parameterization and sensitivity analysis

Several assumptions must be made to apply the MTE to estimate the relative energy use of parasites in comparison to host energy intake. Estimates of the parasite mass scaling exponent (*α*), parasite metabolic activation energy (*E*_a_), and the parasite metabolic normalization constant (*i*) are difficult to obtain empirically and will influence the estimated amount of energy that parasites take from their hosts (Table 1). To determine the consequences of variation within each MTE equation parameter (Equation 1, Table 1) on our estimate of parasite energy flux, a sensitivity analysis was conducted, similar to that of Molnár et al. (2017). Estimates were obtained of *α*, *E*_a_, and *i* from the literature (Table 2). Estimates of fish mass, parasite mass, and lake temperature were obtained from the Lake Aleknagik dataset (Appendix S1).

**FIGURE 1:**
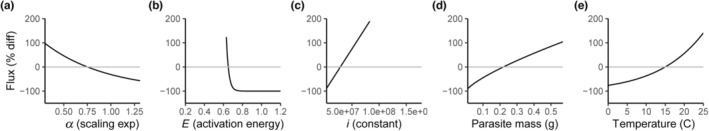
Sensitivity analysis of *Schistocephalus solidus* energy flux across plausible parameter values, measured as percent difference in flux from standard parameter value (Table 2).

The effect of perturbing each parameter value within the previously reported range of each parameter was tested. The resulting estimates of energy flux were compared to those produced by using the “standard” parameter values commonly used in MTE studies (Hechinger, 2013) (Figure 1). This allowed visualization of the sensitivity of energy flux to variation in each parameter value, an important piece of information for accurate calculation of species‐, population‐, and individual‐level energy fluxes. Additionally, interacting parameter values were tested, allowing investigation of how changes in one parameter value altered the influence of another parameter value on the shape of the energy flux curve (Figure S2).

To further investigate sensitivity, parameters derived from the respirometry experiment (*α* and *i* for stickleback) were perturbed from their estimated values. We chose to include only *α* and *i* in this sensitivity experiment, as these were the parameters that we directly estimated in this study. First the mean value of energy flux was calculated using the masses of the Lake Aleknagik stickleback, with *α* and the normalization constant set to the lab‐estimated values (*α* = 1.039, *i* = 17.71). Next, each value was perturbed independently by 10% (i.e., *α*_low_ = .9351 and *α*_high_ = 1.1429, *i*_low_ = 15.939 and *i*_high_ = 19.481). Finally, the percent difference between the lab‐estimated flux calculation and the perturbed‐values flux calculation was determined.

### 2.5 Impact of size variability on MTE‐derived estimates of energy flux

Parasite sizes can vary greatly with respect to their life stage and host, and intraspecific variation is still not well described (e.g., Poulin & Morand, 1997). The observations in this study indicated that the mass of individual *S. solidus* varied considerably, even within individual hosts (e.g., Heins et al., 2002). Since multiple smaller parasites can extract more energy from their host than one large parasite (Hechinger, 2013), it is important to know the potential size distribution of individual parasites within the host. Unfortunately, many datasets only include parasite abundance within each host. This was addressed using a two‐pronged approach. First, all analyses were run on a subset of the Lake Aleknagik data that included only single infections (*n* = 460). Second, another dataset of *S. solidus*‐infected stickleback from Lake Iliamna (*n* = 222) was used to estimate the effect on energy flux calculations of using actual body weight values versus assuming all parasites have body weights equal to the mean of all parasite body weights. The Lake Iliamna fish were collected opportunistically, and thus could not be used for measuring prevalence of *Schistocephalus* infection, but can reasonably be used to quantify size variability of individual *Schistocephalus* within fish infected by multiple worms. For each stickleback, wet mass and standard length were recorded. *S. solidus* were removed from the body cavity using dissecting scissors and forceps. The number of *S. solidus* per stickleback was recorded, and each individual *S. solidus* was weighed, and total length and height (at the widest point of the body) were measured.

**FIGURE 2:**
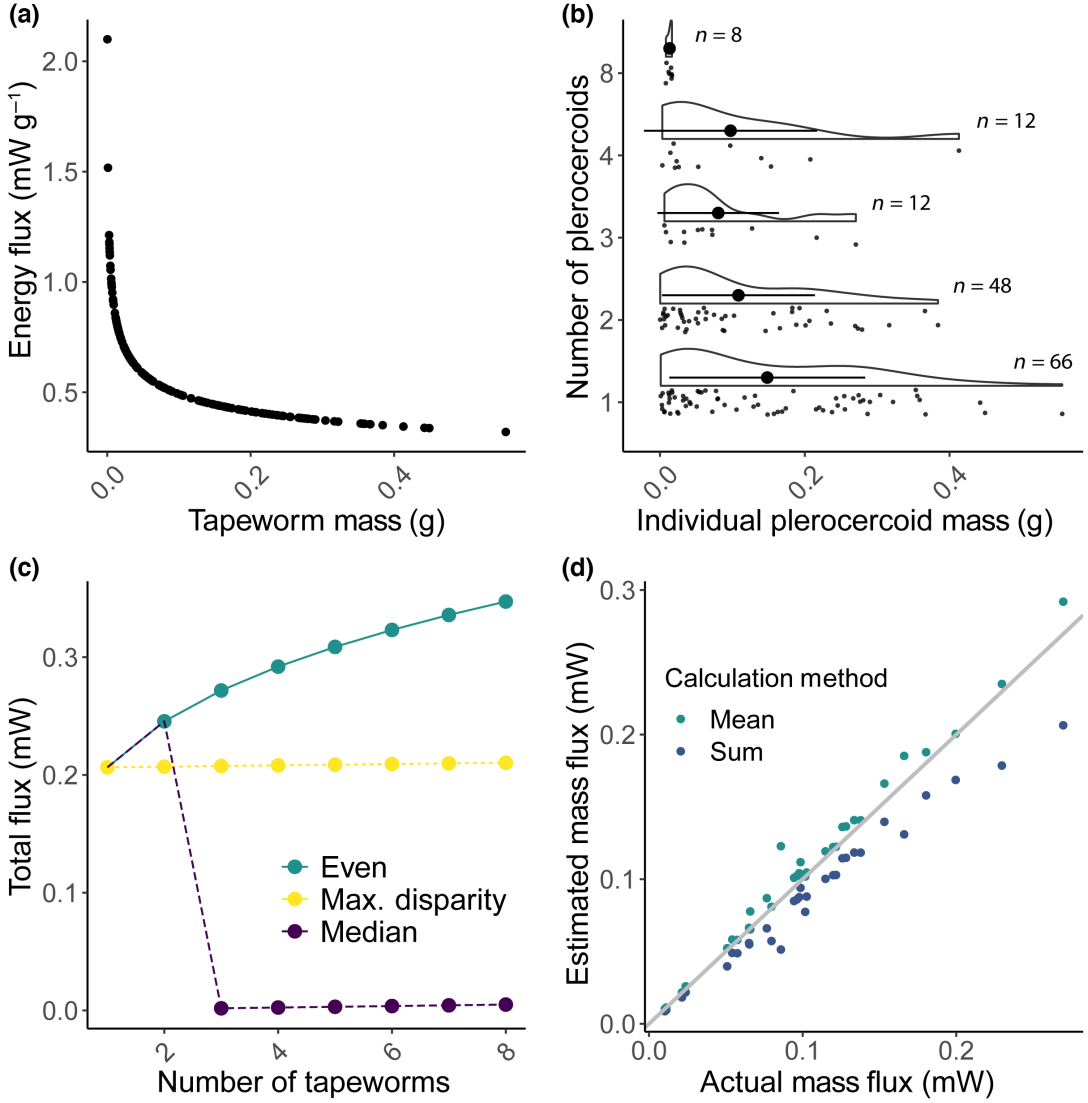
(a) Predicted flux by mass, measured in milliwatts per gram (note, mW g^−1^ = W kg^−1^). Smaller parasites metabolize more per gram than larger parasites when energy flux is calculated using the MTE flux equation. This figure uses weights of individual tapeworms from the Lake Iliamna dataset. (b) The size distributions of individual parasites within single and multiple infections from the Lake Iliamna dataset, showing that size tends to be not normally distributed (*W* = 0.847, *p* < .001). The distribution of the data is visualized, with the mean as a solid black dot and the standard deviation of the mean indicated by whiskers. Rain cloud plots show variability of *Schistocephalus solidus* size within each host. The *y*‐axis represents observed infrapopulation counts, (i.e., *n* = 1, 2, 3, 4, or 8). (c) Assuming mean size within multiple infections leads to higher estimates of flux than assuming differential distributions (“max disparity”; see Section 2), while median estimations underestimate potential flux. This plot shows simulated data bounded by maximum total *S. solidus* weight from the Lake Iliamna dataset. (d) Lake Iliamna energy flux across estimates considering the actual size of each worm in multiple infections (*x*‐axis) vs. energy flux calculated from the sum of all *S. solidus* weights considered as a single individual (e.g., historical datasets that include only the total weight of all parasites, rather than individual parasite weights; in blue), and equal weights of all parasites in multiple infections (i.e., assuming even weights across individuals; in turquoise).

Mean parasite body weight is sometimes used to measure energy flux, but the size distribution of *S. solidus* individuals for both Lake Iliamna and Lake Aleknagik was not normal (Shapiro–Wilk: Lake Iliamna fish *W* = 0.759, *p* < .001; Lake Aleknagik fish *W* = 0.850, *p* < .001). To determine how the use of median or mean mass might over‐ or under‐estimate the flux due to parasites, a theoretical dataset was generated based on the maximum total weight of *S. solidus* within the Lake Iliamna dataset (0.6778 g). Then, a data set of maximum disparity was generated, where all *S. solidus* in multiple infections were assumed to weigh the minimum weight of a single *S. solidus* in the dataset (0.0003 g) except one *S. solidus* in the infection which was assumed to weigh the remainder of the weight (e.g., for dual infections, one worm was 0.0003 g, one was 0.6775 g; for triple infections, two worms were assumed to each weigh 0.0003 g, one was 0.6772 g). The energy flux of the theoretical parasite masses for this maximum disparity dataset was estimated (Figure 2). Energy flux was also estimated based on the assumption that all worms in a fish weighed the same (Figure 2, “Even”) and on the assumption that all worms in a fish weigh the median weight (Figure 2, “Median”).

## 3 RESULTS

### 3.1 Empirical determination of host metabolic rate using respirometry

**FIGURE 3:**
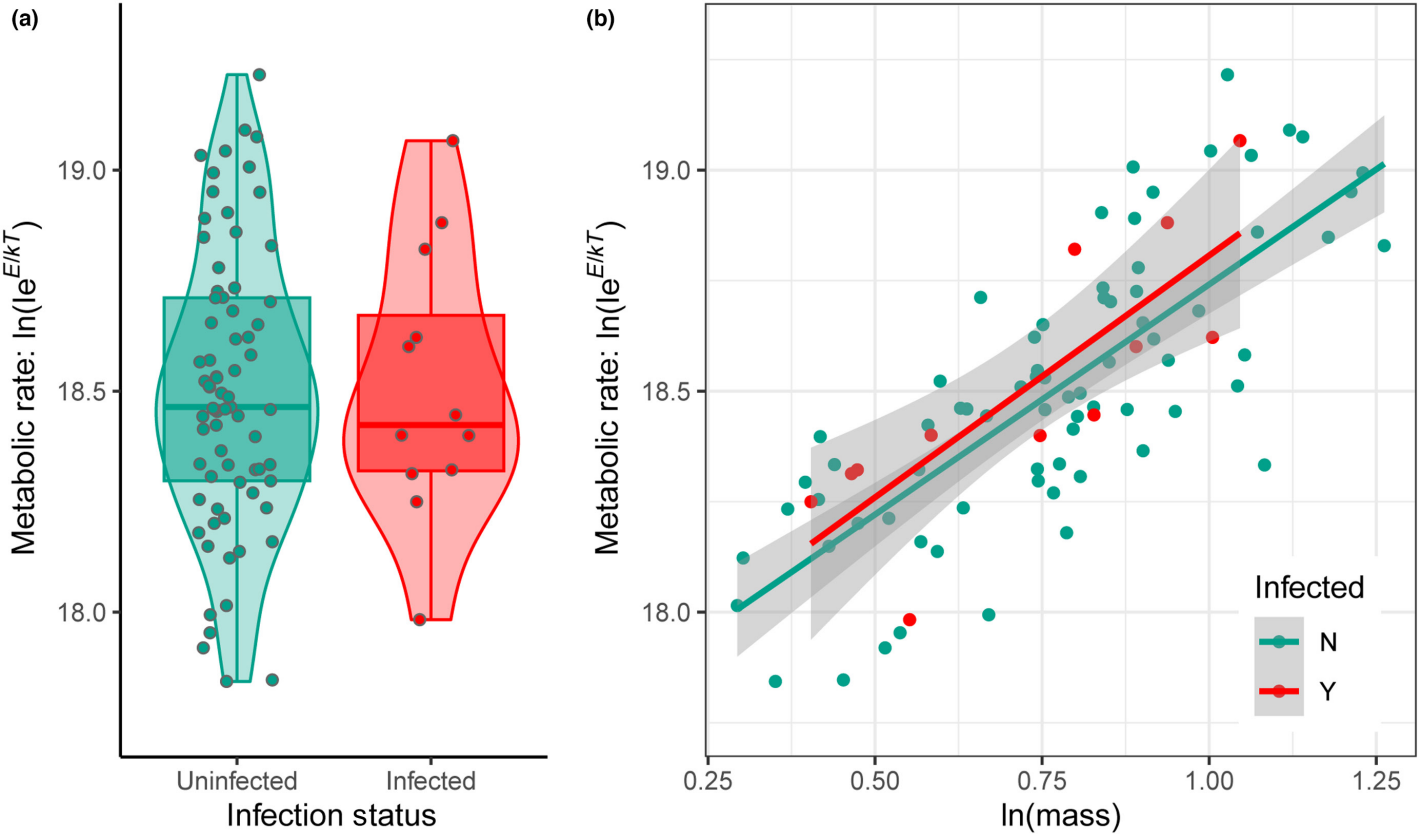
Empirical estimation of metabolic theory of ecology parameters using respiration data (slope = scaling exponent, *α*; intercept = normalization constant, C = ln(*i*)). The *y*‐axis represents the log of temperature‐corrected individual metabolic rate. (a) Temperature‐corrected respiration rate for infected (*n* = 12) vs. uninfected (*n* = 73) three‐spined stickleback. (b) Linear regression models showing how metabolic rate depends on an interaction between host mass and infection status. Each point represents the respiration rate of an individual fish.

Two parameters of the MTE flux equation were estimated by modeling the lab‐measured host respiration rate as a function of host mass: *α* (the model estimate of slope) and the normalization constant (the model estimate of the intercept). The empirically measured mass‐corrected resting respiration rate of uninfected stickleback hosts was 126.5 ± 25.1 mg O_2_ kg^−1^ h^−1^ (mean ± SD), and whole‐organism resting respiration rate of uninfected hosts was 281.6 ± 91.2 mg O_2_ kg^−1^ h^−1^ (mean ± SD) (Table S3). For uninfected stickleback (linear model, infected fish only; mass‐corrected respiration rate ~ mass), the slope of the fitted regression (i.e., *α*) was 1.04 ± .101 and the intercept (i.e., *i*) was 17.70 ± 0.081 (Figure 3; $R_{\mathrm{adj}}^{2}$ = .60, *F*_1,71_ = 106.5, *p* < .001). For infected stickleback (linear model, infected fish only; mass‐corrected respiration rate ~ mass), *α* was 1.09 ± .25 and the intercept was 17.71 ± 0.19 (Figure 3; $R_{\mathrm{adj}}^{2}$ = .66, *F*_1,10_ = 19.0, *p* = .0014). In the full model (mass‐corrected respiration rate ~ host population + infection status + mass), neither host population nor infection status had significant effect (*p* > .05), but the intercept (17.71 ± 0.10) and mass (1.04 ± 0.11) were significant ($R_{\mathrm{adj}}^{2}$ = .59, *F*_3,81_ = 41.7, *p* < .001). When the dataset was randomly subsampled to a balanced design (i.e., *n* = 12 for both infected and uninfected fish), the results were similar with no effect of host population or infection status, but a significant effect of intercept and mass.

### 3.2 Estimating energy flux of *Schistocephalus solidus* in Alaskan sticklebacks

#### 3.2.1 Energy flux of *Schistocephalus solidus*

**FIGURE 4:**
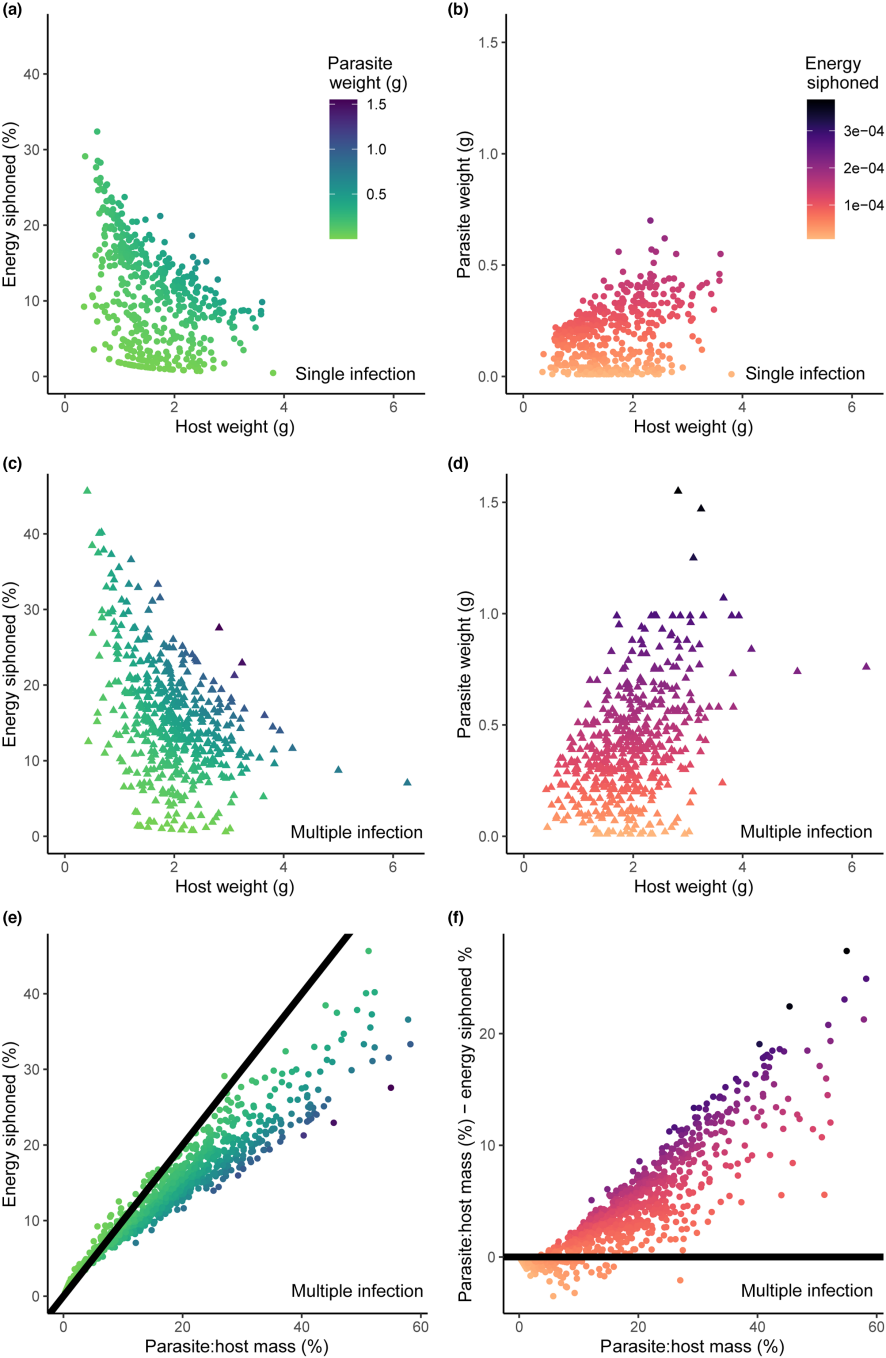
Energy siphoning by *Schistocephalus solidus* (tapeworm parasite) from *Gasterosteus aculeatus* (three‐spined stickleback host) from the Lake Aleknagik dataset, estimated using the metabolic theory of ecology energy flux equation (Equation 1). (a, c) Percentage of host energy siphoned by the parasite across host weights, colored by parasite weight (in panels a and b, total weight = individual weight of a single infection, in panels c and d, total weight = sum of infrapopulation weight, i.e., the sum of all individual weights). (b, d) Energy siphoned (J, in color) plotted by host and parasite weight. (e) Comparison of parasite‐to‐host mass (in percent parasite mass of host mass) with energy siphoning (in percent parasite energy flux of host energy flux). The black line indicates a 1:1 ratio. (f) Difference in mass and energy flux ratios across a range of mass ratios, showing that at low parasite: host mass ratios, mass underestimates energy flux, while at high parasite: host mass ratios, mass overestimates energy flux. Horizontal black line represents equity. Panels a and b represent hosts with a single parasite infection, and panels c–f represent multiple parasite infections (i.e., two or more parasites per host).

Energy siphoning from fish in the Lake Aleknagik field dataset ranged up to 32.4% in single infections and 45.7% in multiple infections (Figure 4a,c). Since multiple infections were reported as a single weight, and since based on theory multiple smaller individuals will have a greater energy flux per gram than a single individual of the same weight, the estimate of energy siphoned from hosts with multiple infections was almost certainly underestimated. The mean percent of host energy siphoned by parasites was 13% ± 8%, when calculated by averaging individual energy siphoning by host. Modeled percent of host energy siphoned by parasites (i.e., slope from $F_{p}\sim \text{slope}*R_{h}$) was 7.8% ± 0.5% (*p* < .001, adjusted *R*‐squared = .20). Flux estimates ranged from 8.74 × 10^−6^ to 2.11 × 10^−4^ watts in single infections (mean ± SD: 7.48 × 10^−5^ ± 4.40 × 10^−5^ watts) and from 8.74 × 10^−6^ to 3.84 × 10^−4^ watts in multiple infections (mean ± SD: 1.35 × 10^−4^ ± 6.49 × 10^−5^ watts) (Figure 4b,d). Parasite‐to‐host mass ratios from fish in the Lake Aleknagik dataset ranged up to 37.3% in single infections (mean ± SD: 12.6 ± 8.5) and 58.2% in multiple infections (mean ± SD: 21.6 ± 12.0) (Figure 4b). The percentage of energy siphoned decreased as parasite‐to‐host mass percent increased (Figure 4e). Furthermore, at low parasite‐to‐host mass ratios, mass underestimates energy flux, while at high parasite‐to‐host mass ratios, mass overestimates energy flux (Figure 4f).

### 3.3 Parameterization and sensitivity analysis

Energy flux estimates vary greatly when model parameters (Table 1) are varied across a range of plausible values (Figure 1). Activation energy (*E*) was the most sensitive parameter, increasing precipitously at values smaller than the standard range (i.e., ~0.63, Brown et al., 2004; Figure 1b).

The shape of the energy flux curve also depended on statistical interactions between parameters (Figure S2). For example, at low mass values, the scaling exponent (*α*) can have a greater absolute effect on flux than at high mass values (Figure S2a,d,g). Varying one parameter can also change the shape of the curve across the range of another parameter. For example, at low scaling exponents (i.e., hypoallometric scaling), the parameter curve for mass is saturating, while at high scaling exponents (i.e., hyperallometric scaling), the parameter curve for mass is exponential (Figure S2b,e,h).

When lab‐estimated parameter values *α* and *i* were perturbed by 10%, estimated energy flux varied. When *α* was decreased by 10%, energy flux increased by 7%, and when it was increased by 10%, energy flux decreased by 7%. When *i* was decreased by 10%, energy flux increased by 83%, and when *i* was increased by 10%, energy flux decreased by 488%.

### 3.4 Impact of size variability on MTE‐derived estimates of energy flux

Simulations of systematic variation in individual parasite size, which is the natural case for *S. solidus* cestodes, illustrate how energy flux is biased by replacing individual parasite weights with the mean parasite weight. As predicted by general empirical scaling patterns of multicellular life, small parasites metabolize more per gram of body mass than do larger parasites (Hechinger et al., 2012; Hechinger, 2013; Figure 2a). Additionally, the weight distribution of *S. solidus* is right skewed; that is, most individuals weigh less than the mean (Figure 2b). When *S. solidus* energy flux was calculated using the simulated dataset at a constant total weight (0.6778 g), results showed that assuming an even weight distribution (e.g., in double infections, each *S. solidus* weighed 0.3389 g; in triple infections, 0.2259 g), overestimated energy flux considerably (Figure 2c) compared to assuming an uneven weight distribution (e.g., in double infections, *S. solidus* weighed 0.6775 and 0.0003 g).

Empirical calculations of energy flux, based on multi‐year *S. solidus* size frequency distribution in a single lake, provide a realistic conservative estimate of how much MTE parameter choice biases interpretations. If the average weight is assumed for each individual *S. solidus*, energy flux is overestimated by up to 43% (mean ± SD = 6.19% ± 8.12%; Figure 2c). Conversely, if the sum of *S. solidus* weights in a host are assumed to be combined into one *S. solidus*, energy flux is underestimated by up to 40.21% (mean ± SD = 15.00% ± 7.32%).

## 4 DISCUSSION

This study aimed to quantify the magnitude and sensitivity of energy flux estimates for stickleback hosts and tapeworm parasites, following Hechinger's (2013) approach to the metabolic theory of ecology (Brown et al., 2004). Individual *S. solidus* tapeworms can extract up to ~32% of their host's baseline energy requirement, while multiple infections can extract up to ~45% of the host's baseline energy requirement. While these results are promising, we also ran into several potential pitfalls that could skew estimates of energy flux in similar studies. These pitfalls are discussed below so that other researchers can work to apply MTE to parasite energy flux in their focal systems.

Estimates of respiration rate in uninfected individuals (126.5 ± 25.1 mg O_2_ kg^−1^ h^−1^, mean ± SD; masses reported as wet mass; mass = 2.22 ± 0.52 g [mean ± SD]; Table S3) were within the range of previously measured three‐spined stickleback respiration rates (Tudorache et al., 2007). Previous comparisons of respiration rate between infected and uninfected individuals have been equivocal, with one study showing a higher respiration rate in infected individuals (Andrea et al., 2017) and another that found very little difference between infected and uninfected individuals (Lester, 1971). In the present study, no significant difference in mean temperature‐corrected individual metabolic rate was found between uninfected and infected individuals. The lack of a significant difference between uninfected and infected fish in this study, as well as the equivocal findings of prior studies, may be because this difference is relatively small. Additionally, worms in this study were relatively small, and additional measurements of more heavily infected fish may reveal differences in respiration rate between infected and uninfected fish. Alternatively, since host organisms partition resources to different functions (e.g., growth, reproduction, activity), the worms could be siphoning resources that the stickleback would otherwise invest in growth or reserves without necessitating an increase in the metabolic rate of the fish (e.g., Barber, 2007). Furthermore, since hosts with lower metabolic rates have a greater fraction of their energy allocated to parasites (Grunberg & Anderson, 2022), retaining a lower metabolic rate even when infected may limit both host losses and parasite infrapopulation growth.

When respiration rates were used to estimate MTE energy flux parameters *i* and *α* (i.e., the normalization constant and the allometric scaling exponent), results were relatively consistent between infected and uninfected fish (Figure 3). The normalization constants that were estimated for three‐spined stickleback were lower than the estimate for fish reported in Brown et al. (2004). As noted in Hirt et al. (2017), the normalization constant accounts for a wide variety of potential influences, including taxon‐specific effects, changes in overall metabolic state due to season or life stage, and the type of metabolism occurring (e.g., aerobic vs. anaerobic). Disentangling the specific effects of each of these influences will require additional experiments; future work focused on the consequences of these influences would allow for a more comprehensive understanding of the normalization constant and its predictability. The observed *α* was higher than the theoretical value of ¾, but within the range of previous values (Ehnes et al., 2011; White et al., 2007). For estimates of both the normalization constant and the mass‐scaling constant, the known presence of parasites within the stickleback population may partially explain the deviations in our data from prior observed values (Hechinger et al., 2019). Our results highlight the need for direct estimates of parasite metabolism to accurately parameterize models of host and parasite respiration, due to the fact that parasites both metabolize at different basal rates than their hosts (Von Brand, 1973) and can alter host metabolism itself (Walkey & Meakins, 1970).

This study conservatively estimates that individual *S. solidus* parasites might be siphoning up to 32% of their host's baseline energy requirement, increasing to 46% in multiply infected fish. In the Lake Aleknagik dataset, mean individual energy siphoning and modeled energy siphoning were largely congruent at 13% (±8%) and 7.8% (±0.5%), respectively. These estimates are sensitive to MTE energy flux parameters, so we put these numbers forward as a starting point for further validation and improvement. A logical next step will be to measure respiration of *S. solidus*, although this must be done carefully, as measurements of *S. solidus* taken within the host could be conflated with host‐by‐parasite interactions (i.e., the respiration of an infected host is likely not simply additive of host + parasite), and measures of *S. solidus* taken outside the host could be conflated by parasite behavior (i.e., ex situ respiration may vary significantly from in situ rates). Although previous studies have measured *S. solidus* respiration (Davies & Walkey, 1966), these estimates stand to be updated with modern respirometry measurement systems. Another consideration for future research is determination of how worms interact and influence respiration of both fish and conspecifics in multiple infections. Since *S. solidus* are parasitic castrators (Kuris, 2003; Lafferty & Kuris, 2002), smaller worms in multiple infections may be suppressed, which could mitigate the fact that smaller worms have a higher energy flux per gram compared to larger individuals. Conducting additional respirometry experiments across various host and parasite taxa will also help to further our understanding of patterns of variability in empirical MTE energy flux parameters across phylogeny.

Sensitivity analyses illustrate that energy flux estimates are highly sensitive to parameter selection, with activation energy (*E*) being particularly influential around the lower edge of its range, and the normalization constant (*i*) being strongly influential across probable values (Figure 1). For *E*, the steepest slope was around the expected value most frequently used in the literature, implying that small variations in E could cause large changes in estimated energy flux. However, average activation energy is fairly consistent for rate‐limiting enzyme‐catalyzed biochemical reactions of aerobic metabolism across organisms Gillooly et al., 2001, and this consistency may mitigate the practical effect of this extreme sensitivity. On the other hand, variability of normalization constants among species can cause incorrect estimates of energy flux from host to parasite. Additionally, because the normalization constant (*i*) is derived from the slope of temperature‐corrected respiration across body mass (Brown et al., 2004), where the slope, C = ln(*i*), small changes in C are amplified since *i* = e^C^. For the Lake Aleknagik data set, perturbing *i* by ±10% caused a −488% to 83% difference in energy flux compared to energy flux calculated using lab‐estimated parameters, further emphasizing the sensitivity of this parameter. Furthermore, the normalization constant must be derived empirically, as there are not yet any general rules defining how it varies across taxa. We expect that this will be the greatest research opportunity to address for broad application of the MTE for standardizing estimates of energy flux from hosts to parasites. We encourage researchers to conduct sensitivity analyses (as in Figure 1) over feasible parameter values in their systems (e.g., Ehnes et al., 2011; Uyeda et al., 2017; White et al., 2019) and, importantly, to further investigate whether there are any general rules governing the normalization constant across organisms and ecosystems.

Although many studies assume that all parasites in multiple infections are the same mass or equal to the mean parasite mass, this study shows that this assumption can overestimate total energy transfer. This overestimation of flux is particularly acute when parasites display a skewed mass distribution, as is often the case for *S. solidus* and many other parasite taxa. This is due to Jensen's inequality or the “fallacy of the average,” wherein the performance of an organism under average conditions is typically not equal to performance averaged across a range of conditions (Denny, 2017). These results expand on the theoretical framework by Price et al. (2012), who pointed out that if temperature or activation energies differ, metabolic rate would be only an approximation because of the problem of averaging nonlinear functions. Specifically, this study reiterates previous work showing that energy flux calculated using the mean mass is not equal to mean respiration at different masses (Equation 4, where <> indicates mean values; Figure 2; Savage, 2004; White et al., 2007).

(4) $$i{<M}_{x}^{\alpha_{x}}>e^{-E/\mathrm{kT}}\neq <iM_{x}^{\alpha_{x}}e^{-E/\mathrm{kT}}>$$

Whether or not population‐level mean parasite size can be used for flux calculations is an important consideration for studies attempting to quantify siphoning of individual host energy flux based on parasite abundance. For example, if all parasites of a species are essentially the same size, then it would be possible to measure a subset of parasites, and then calculate flux based on their mean body size and total abundance, resulting in substantial time savings. Conversely, for parasites with extreme variability in body size (e.g., *S. solidus*), measuring each parasite will be important for accurate energy flux estimates. If this is not feasible, energy flux estimates can be bounded by finding (1) an upper bound by assuming mean parasite size for each parasite and (2) a lower bound by assuming a single infection (comprising the entire weight of all parasites), with the caveat that both of these bounds will be wrong, but that the correct answer will lie somewhere in between. Alternatively, it might be possible to measure a representative subset of the sampled parasites and estimate a size distribution, which could then be used to determine the magnitude of underestimation. The feasibility and potential accuracy of this approach remains to be tested.

MTE‐produced calculations of energy flux from host to parasite provide inherently conservative estimates of impacts on hosts, because these calculations do not consider any other mechanisms by which parasites could increase or decrease host metabolism (Hechinger et al., 2012; Morand & Harvey, 2000; Walkey & Meakins, 1970), nor do they consider host energy expenditures on immune function or parasite‐induced behavioral change (Makrinos & Bowden, 2016; Scharsack et al., 2016, 2021). Additionally, these estimations do not include a constant to account for resource‐supply conversion efficiency (noted in Hechinger, 2013), which is variable both within and among species (Sanders et al., 2016). Furthermore, these calculations assume that oxidative respiration is a proxy for total respiration in the system, although many parasites use a substantial amount of anaerobic respiration compared to free‐living organisms (Hechinger et al., 2012). Therefore, these calculations are merely an estimate of energy lost due to parasite metabolism, and thus are almost certainly an underestimate of total host energy lost to parasitism.

The amount of energy siphoned from stickleback by tapeworms is large and emphasizes the importance of future work focusing on parasite components of ecosystem energetics. The approach of using the MTE to calculate energy flux provides great promise as a quantitative foundation for such estimates and provides a more concrete metric of parasite impact on hosts than numerical parasite burden alone (Hechinger et al., 2019). However, the application of metabolic scaling parameters that were originally derived from broad‐scale interactions (e.g., Brown et al., 2004) to local interactions (e.g., stickleback and *S. solidus*) requires consideration of error propagation. Further quantifying sensitivity to parameter choices and potential error propagation represents a key research focus before this approach can be applied to a wide range of host–parasite systems. First, and perhaps foremost, unless standardized normalization constants can be empirically determined, analysts must use caution when making estimates of the proportion of a host's energy siphoned by its parasite(s). Second, if parasite mass is variable, caution must be taken to ensure that using population‐level or species‐level mean parasite mass does not skew energy flux calculations. Finally, this study focused on a single parasite species, and for wide application across systems, it will be necessary to assess energy flux of multiple species of parasite within each host species. Therefore, we anticipate that, with careful consideration of sensitivity and the incorporation of additional empirical data, the MTE could be widely used to compare the energetic impacts of parasitism across organisms and ecosystems.

## AUTHOR CONTRIBUTIONS

**Danielle C. Claar:** Conceptualization (equal); data curation (equal); formal analysis (lead); methodology (equal); visualization (lead); writing – original draft (lead); writing – review and editing (equal). **Sara M. Faiad:** Conceptualization (equal); formal analysis (equal); investigation (equal); methodology (equal); writing – review and editing (equal). **Natalie C. Mastick:** Conceptualization (equal); formal analysis (equal); investigation (equal); methodology (equal); writing – review and editing (equal). **Rachel L. Welicky:** Conceptualization (equal); formal analysis (equal); investigation (equal); methodology (equal); writing – review and editing (equal). **Maureen A. Williams:** Conceptualization (equal); formal analysis (equal); investigation (equal); methodology (equal); writing – review and editing (equal). **Kristofer T. Sasser:** Conceptualization (equal); data curation (equal); formal analysis (equal); investigation (equal); methodology (equal); writing – review and editing (equal). **Jesse N. Weber:** Conceptualization (equal); funding acquisition (equal); methodology (equal); project administration (equal); resources (equal); supervision (equal); writing – review and editing (equal). **Chelsea L. Wood:** Conceptualization (equal); formal analysis (equal); investigation (equal); methodology (equal); supervision (equal); validation (equal); writing – review and editing (equal).

## CONFLICT OF INTEREST STATEMENT

The authors declare no conflicts of interest.

## Supporting information


Appendix S1.

## Data Availability

The data and code that support the findings of this study are openly available at https://github.com/wood‐lab/Claar_etal_MTE_EcolEvol.
