# Supplementary material for: Estimating the magnitude and sensitivity of energy fluxes for stickleback hosts and Schistocephalus solidus parasites using the metabolic theory of ecology
Source: Ecol Evol. 2023 Dec 3;13(12):e10755. doi: 10.1002/ece3.10755 (PMC10694383; doi:10.1002/ece3.10755)
Supplement: Supplementary file 1 — Appendix S1. [file ECE3-13-e10755-s001.docx]

# Supplemental Information

## Supplementary Tables

**Table S1.** Sample size of Lake Aleknagik fish dataset by site

| **Coordinate location** | **Sample Site Name** | **n** |
| --- | --- | --- |
| 441 | Deep Off Bear Bay Pt. Aleknagik | 75 |
| 439 | Deep Off Bear Bay Pt. Aleknagik (2) | 90 |
| 840 | Mouth of Hansen's Creek, Aleknagik | 280 |
| 816 | Off Agulowak R., Aleknagik | 50 |
| 440 | Off Bear Bay Pt., Aleknagik | 60 |
| 442 | Off Bear Bay Pt. Aleknagik (2) | 60 |
| 346 | Off Whitefish Pt., Aleknagik | 35 |
| 345 | Off Whitefish Pt., Aleknagik (2) | 26 |
| 340 | Picnic Beach (Boar Bay Pt), Aleknagik | 60 |
| 647 | Picnic Island, Aleknagik | 255 |
| 750 | Pike Bay, Aleknagik | 181 |
| 849 | Pike Bay, Aleknagik (2) | 229 |
| ULA | Upper end of Aleknagik | 202 |
| 145 | W. of Yako Cr., Aleknagik | 60 |
| 438 | W. of Bear Bay Pt | 60 |
| 247 | Whitefish Pt | 60 |
|  | *Total* | 1783 |

**Table S2.** Sample sizes by host ‘population’. GS = F/M hybrid of Gosling/Sayward; SG = F/M hybrid of Sayward/Gosling. Fish were exposed and measured in two rounds (September 2020 and December 2020). Numbers show initial sample size, and numbers in parentheses show the number of fish for which respiration rate was successfully measured (excluded samples represent bubbles in the chamber, or malfunctioning equipment).

|  | Not exposed - Sept | Not exposed - Dec | Total - Not Exposed | Exposed - Sept | Exposed - Dec | Total - Exposed |
| --- | --- | --- | --- | --- | --- | --- |
| GS | 8 (6) | 2 (2) | 10 (8) | 32 (27) | 5 (4) | 37 (31) |
| SG | 8 (6) | 2 (2) | 10 (8) | 32 (26) | 13 (12) | 45 (38) |
| Total | 16 (12) | 4 (4) | 20 (16) | 64 (53) | 18 (16) | 82 (69) |

**Table S3.** Mass, mass-corrected respiration, and whole-organism respiration for uninfected fish from laboratory experiment

|  |  | GS  (n = 27) | SG  (n = 26) | Both populations (pooled) |
| --- | --- | --- | --- | --- |
| Mass (g) | mean ± s.d. | 2.46 ± 0.454 | 2.01 ± 0.485 | 2.22 ± 0.519 |
|  | min | 1.88 | 1.34 | 1.34 |
|  | max | 3.53 | 3.25 | 3.25 |
| Mass-corrected respiration  (mg O_2_ kg^-1^ hr^-1^) | mean ± s.d. | 133 ± 25 | 132 ± 24 | 127 ± 25 |
|  | min | 111 | 92 | 77 |
|  | max | 166 | 166 | 196 |
| Whole-organism respiration (mg O_2_ hr^-1^) | mean ± s.d. | 315 ± 87 | 253 ± 86 | 282 ± 91 |
|  | min | 161 | 139 | 139 |
|  | max | 483 | 547 | 547 |

##

## Supplementary Figures


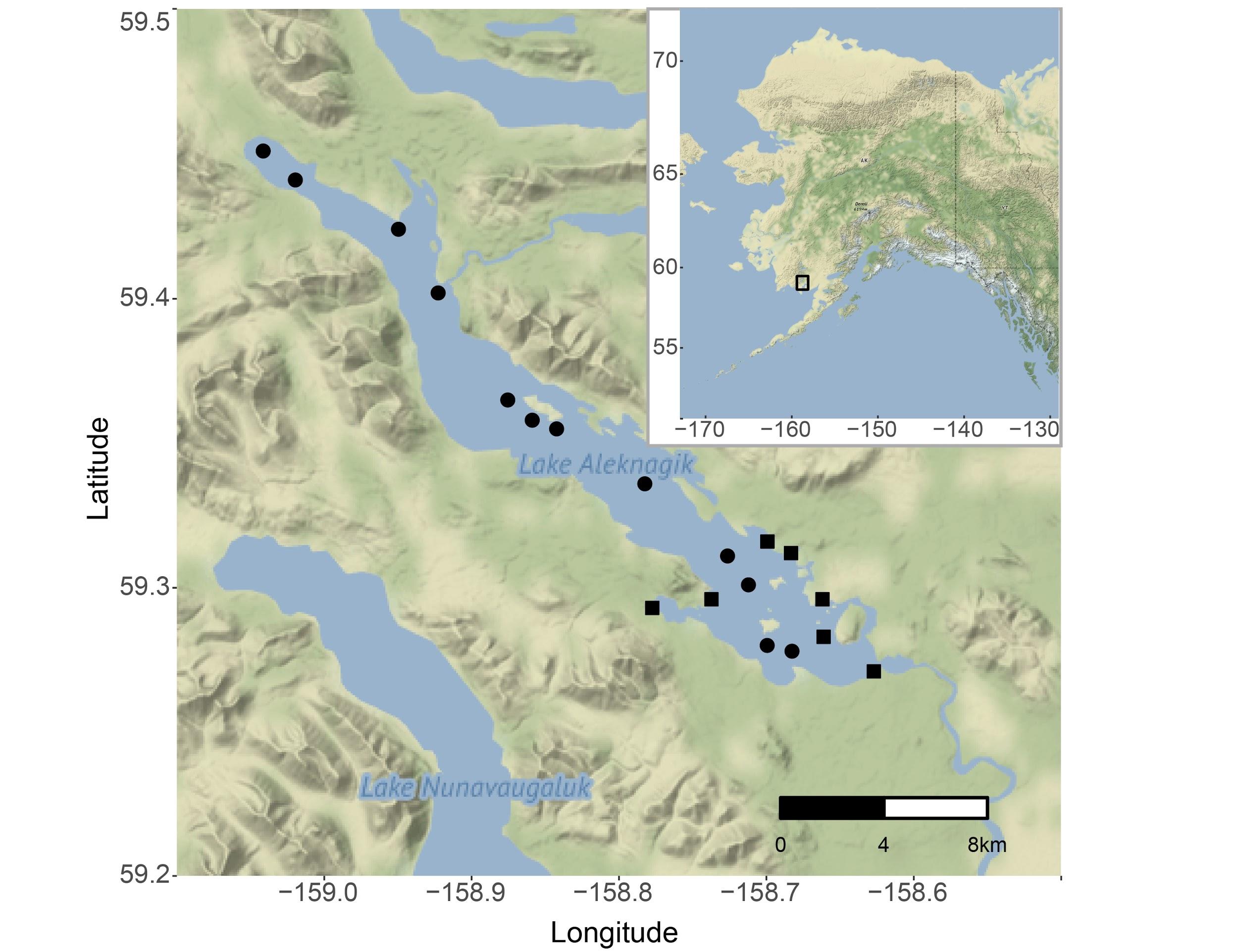


**Figure S1.** Map of Lake Aleknagik sample sites. Squares represent littoral sites, and circles represent limnetic sites.


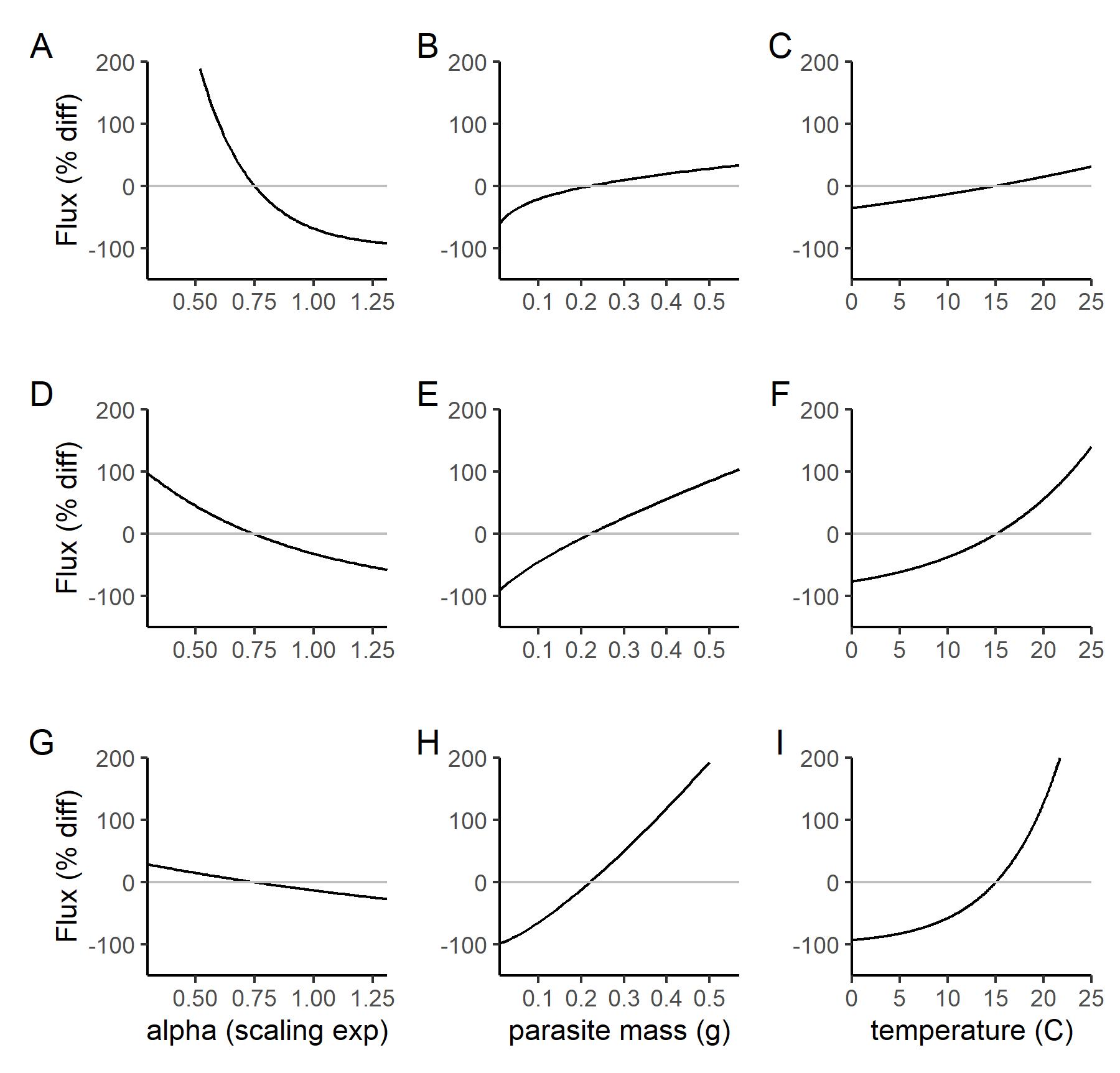


**Figure S2.** Sensitivity analysis indicating outcome of interactions among varying parameter values. Left column is the effect of mass on alpha, the middle column is the effect of alpha on mass, and the right column is the effect of E on temperature. The top row is parameterized with the minimum value of the parameter of interest (i.e., left to right: mass, alpha, E), middle row is parameterized with the standard or mean value of the parameter of interest, and the bottom row is parameterized with the maximum value of the parameter of interest.


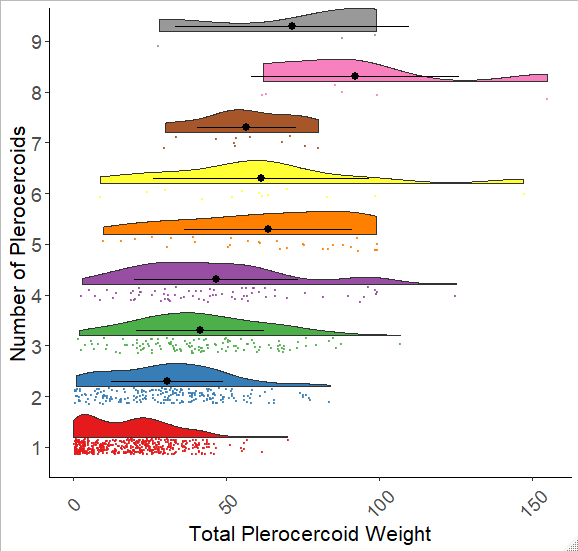


**Figure S3.** Weight distribution of multiple infections in Lake Aleknagik, showing summed *S. solidus* weight.

## Supplementary Methods and Results

### Lake Aleknagik dataset description

Data were collected in Lake Aleknagik (Wood River Lakes, Alaska, USA) during the summers of 1960 and 1961 by Don Rogers, and original data sheets were obtained from the University of Washington, Alaska Salmon Program archive (unpublished data). Sticklebacks were collected using townets and beach seines at 16 sites around Lake Aleknagik (59.3°N, 158.8°W; Table S1). Length (mm), weight (hundredths of a gram), and sex were recorded for each fish, as well as fish color, kidney condition, and stomach contents. Additionally, testes condition or ovary weight, number of ova, ova diameter, and condition were recorded. Each fish was dissected to detect parasite abundance and the number of *S. solidus* individuals and total weight were recorded. Although other parasites were encountered, including *Triaenophorus*, acanthocephalans, nematodes, copepods, and skin cysts, *S. solidus* accounted for the largest proportion of parasite biomass within each fish host, so we only consider *S. solidus* infections in energy flux calculations (i.e., excluding energy flux of other parasite species). Therefore, these flux calculations are a conservative estimate of the amount of energy diverted by the full community of parasites in these fish hosts.

This dataset was composed of 948 infected three-spined sticklebacks containing a total of 1,941 individual *S. solidus* tapeworms. *S. solidus* abundance per infected fish ranged from 1–9 *S. solidus* per infected fish (mean ± s.d.=3 ± 1). *S. solidus* weight ranged from 0.01–0.7 g (mean ± s.d.=0.19 ± 0.14 g) in single infections, and from 0.01–1.55 g (mean ± s.d.=0.30 ± 0.23 g) in all infected fish (i.e., including single and multiple infections). Stickleback weight ranged from 0.37– 7.02 g (mean ± s.d.=2.10 ± 0.85 g), and length ranged from 36–84 mm (mean ± s.d.=61 ± 8.0 mm).

### Lake Iliamna dataset description

The contemporary dataset from Lake Iliamna was composed of 146 infected three-spined sticklebacks, containing a total of 310 individual *S. solidus* tapeworms. An additional 76 sticklebacks were dissected, classified as uninfected, and therefore not included in any further analyses. *S. solidus* abundance per infected fish ranged from 1–8 (mean ± s.d. = 2 ± 2). Individual *S. solidus* weight ranged from 0.0003–0.556 g (mean ± s.d. = 0.117 ± 0.121 g; Figure S3), and length ranged from 7–63 mm (mean ± s.d. = 28 ± 10 mm). Stickleback weight ranged from 0.06–2.66 g (mean ± s.d. = 1.23 ± 0.535 g), and length ranged from 20–64 mm (mean ± s.d. = 50 ± 9 mm).

###

### Infecting British Columbia sticklebacks with *Schistocephalus solidus*

#### Fish husbandry and exposure to *S. solidus*

*G. aculeatus* fish were hatched in June 2019 and two rounds of infections of fish with *S. solidus* occurred. Fish originated from a hybridized ‘population’ of three-spined stickleback comprised of reciprocal crosses of 2 lab-reared stickleback populations; from Gosling Lake (G) and Sayward (S) (British Columbia; 50.385, -125.951) (n=30 per crossed population; “SG” represents female S and male G parentage, and “GS” represents female G and male S parentage). To infect sticklebacks with *S. solidus,* the parasites were first reared by incubating eggs for 2 weeks in 1-mL well plates at ambient temperature (~24°C). Once hatched, coracidia (larval *S. solidus*) were exposed to fasted *Macrocyclops albidus* copepods (n = 40) for 3 weeks in 300-mL open containers. After copepods were exposed to *S. solidus*, the copepods were exposed to the sticklebacks (n_total_=82) for 6 hours in the stickleback’s housings (800-mL containers; 20°C). Sixteen SG and 16 GS fish were exposed on 9/22/20 (tanks SG1 and GS1). Sixteen SG and 16 GS fish were exposed on 9/23/20 (tanks SG2 and GS2). Thirteen SG and 5 GS were exposed on 12/17/20 (tanks SG4 and GS4). One GS fish died after exposures but before measurements; sample sizes are included in Table S2.

#### Experiment Block Design

Sham-exposed fish

*Day 1 (Group 1):* 2 SG unexposed, 2 GS unexp

September Exposure -> 40+ days post exposure

*Day 1 (Group 2):* 1 SG D1 exposed, 2 GS D1 exposed, 1 SG unexposed

*Day 2 (Group 3):* 1 GS D1 exposed, 2 SG D1 exposed, 1 GS unexposed

*Day 3 (Group 4):* 1 SG D1 exposed, 1 GS D1 exposed, 1 SG D2 exposed, 1 GS D2 exposed

*Day 4 (Group 5):* 1 SG D2 exp, 2 GS D2 exp, 1 SG unexposed

*Day 5 (Group 6):* 1 GS D2 exp, 2 SG D2 exp, 1 GS unexposed

September Exposure -> 60+ days post exposure

*Day 1 (Group 7):* 1 SG D1 exp, 2 GS D1 exp, 1 SG unexposed

*Day 2 (Group 8):* 1 GS D1 exp, 2 SG D1 exp, 1 GS unexposed

*Day 3 (Group 9):* 1 SG D1 exp, 1 GS D1 exp, 1 SG D2 exp, 1 GS D2 exp

*Day 4 (Group 10):* 1 SG D2 exp, 2 GS D2 exp, 1 SG unexposed

*Day 5 (Group 11):* 1 GS D2 exp, 2 SG D2 exp, 1 GS unexposed

*Day 6 (Group 12):* 1 SG D1 exp, 1 GS D1 exp, 1 SG D2 exp, 1 GS D2 exp

*Day 7 (Group 13):* 1 SG D1 exp, 1 GS D1 exp, 1 SG D2 exp, 1 GS D2 exp

September Exposure -> 80+ days post exposure

*Day 1 (Group 14):* 2 SG D1 exp, 1 GS D1 exp, 1 GS unexposed

*Day 2 (Group 15):* 2 GS D1 exp, 2 GS D1 exp

*Day 3 (Group 16):* 1 SG D1 exp, 2 GS D1 exp, 1 SG unexposed

*Day 4 (Group 17):* 2 SG D2 exp, 2 GS D2 exp

*Day 5 (Group 18):* 1 SG D2 exp, 2 GS D2 exp, 1 GS unexposed

*Day 6 (Group 19):* 2 SG D2 exp, 2 GS D2 exp

*Day 7 (Group 20):* 2 SG D2 exp, 1 SG unexposed

December Exposure -> 60+ days post exposure

*Day 1 (Group 21):* 2 SG exp, 1 GS exp, 1 GS unexposed

*Day 2 (Group 22):* 3 SG exp, 1 GS exp

*Day 3 (Group 23):* 2 SG exp, 1 GS exp, 1 SG unexposed

*Day 4 (Group 24):* 2 SG exp, 1 GS exp, 1 GS unexposed

*Day 5 (Group 25):* 3 SG exp, 1 GS exp

*Day 6 (Group 26):* 1 SG exp, 1 SG unexposed

### Respirometry protocol

Prior to measurement, fish were fasted for 24 hr. Fish were then placed in individual 330-mL respirometry chambers submerged in a temperature-controlled stock tank for 23 hr. Temperature was maintained at 15°C using a Teco model TK2000H chiller. Submersible pumps (Imagitarium) were used to maintain a constant flow of water within each chamber and an identical pump was used to flush oxygenated water from the stock tank into the chambers intermittently. A total of 32 measurement cycles were recorded for each trial. Each measurement cycle consisted of a 1200 s flush period in which oxygenated tank water flowed into the chambers, a 180 s wait period during which the flow of tank water halted and the chambers were sealed, and a 1200 s measurement period during which the oxygen concentration of each sealed chamber was recorded. The cycling of the flush pump was automated using AquaResp3 [(Svendsen *et al.* 2019)](https://paperpile.com/c/E61Qc0/8Sl5). Oxygen-sensitive REDFLASH contactless sensor spots connected to a PyroScience Firesting O_2_ oxygen meter were used to record the temperature corrected oxygen concentration (mg L^-1^) in each chamber continuously (once per second). Background oxygen consumption was recorded for each chamber prior to and after each trial.

*Calculating background, and absolute and mass specific metabolic rates of fish*

To calculate the background, absolute and mass specific metabolic rates, we used the FishResp package in R [(Morozov *et al.* 2019)](https://paperpile.com/c/E61Qc0/xK46). After importing data on bacterial respiration and raw oxygen unique to each flush cycle and respirometry chamber, we corrected the oxygen data for background respiration using a linear prediction method that uses the pre- and post-oxygen consumption data as the reference points using the function *correct.meas()*. To ensure that the fish were not stressed, and therefore respiring abnormally, we performed a quality control check where we measured the mass-specific metabolic rate before and after correction for background respiration using the function *QC.activity()*. We found no anomalies. Next, we extracted the target slopes to calculate the mass-specific metabolic rate by selecting the three cycles with the lowest metabolic rate for each chamber/fish using the function *extract.slope()*. A linear regression of the extracted slopes was then conducted for each chamber using the function *QC.slope()*. Finally, using these data we calculated the background, absolute, and mass specific metabolic rates using the function *calculate.MR()* (R script available at https://github.com/wood-lab/MTE-stickleback-parasites.
